# Context-Contingent Privacy Concerns and Exploration of the Privacy Paradox in the Age of AI, Augmented Reality, Big Data, and the Internet of Things: Systematic Review

**DOI:** 10.2196/71951

**Published:** 2025-05-14

**Authors:** Christian Herriger, Omar Merlo, Andreas B Eisingerich, Annisa Rizkia Arigayota

**Affiliations:** 1 Imperial Business School Imperial College London London United Kingdom

**Keywords:** privacy paradox, systematic literature review, contextual integrity, artificial intelligence, Internet of Things, privacy concerns

## Abstract

**Background:**

Despite extensive research into technology users’ privacy concerns, a critical gap remains in understanding why individuals adopt different standards for data protection across contexts. The rise of advanced technologies such as the Internet of Things (IoT), artificial intelligence (AI), augmented reality (AR), and big data has created rapidly evolving and complex privacy landscapes. However, privacy is often treated as a static construct, failing to reflect the fluid, context-dependent nature of user concerns. This oversimplification has led to fragmented research, inconsistent findings, and limited capacity to address the nuanced challenges posed by these technologies. Understanding these dynamics is especially crucial in fields such as digital health and informatics, where sensitive data and user trust are central to adoption and ethical innovation.

**Objective:**

This study synthesized existing research on privacy behaviors in emerging technologies, focusing on IoT, AI, AR, and big data. Its primary objectives were to identify the psychological antecedents, outcomes, and theoretical frameworks explaining privacy behavior, and to assess whether insights from traditional online privacy literature, such as e-commerce and social networking, apply to these advanced technologies. It also advocates a context-dependent approach to understanding privacy.

**Methods:**

A systematic review of 179 studies synthesized psychological antecedents, outcomes, and theoretical frameworks related to privacy behaviors in emerging technologies. Following established guidelines and using leading research databases such as ScienceDirect (Elsevier), SAGE, and EBSCO, studies were screened for relevance to privacy behaviors, focus on emerging technologies, and empirical grounding. Methodological details were analyzed to assess the applicability of traditional privacy findings from e-commerce and social networking to today’s advanced technologies.

**Results:**

The systematic review revealed key gaps in the privacy literature on emerging technologies, such as IoT, AI, AR, and big data. Contextual factors, such as data sensitivity, recipient transparency, and transmission principles, were often overlooked, despite their critical role in shaping privacy concerns and behaviors. The findings also showed that theories developed for traditional technologies often fall short in addressing the complexities of modern contexts. By synthesizing psychological antecedents, behavioral outcomes, and theoretical frameworks, this study underscores the need for a context-contingent approach to privacy research.

**Conclusions:**

This study advances understanding of user privacy by emphasizing the critical role of context in data sharing, particularly amid ubiquitous and emerging health technologies. The findings challenge static views of privacy and highlight the need for tailored frameworks that reflect dynamic, context-dependent behaviors. Practical implications include guiding health care providers, policy makers, and technology developers toward context-sensitive strategies that build trust, enhance data protection, and support ethical digital health innovation.

**Trial Registration:**

PROSPERO CRD420251037954; https://www.crd.york.ac.uk/PROSPERO/view/CRD420251037954

## Introduction

### Technological Advances and the Evolving Privacy Landscape

The context in which data are collected is crucial to individuals, as they are more likely to feel concerned about their privacy when their conversations are recorded without their knowledge or consent [1]. Moreover, the latest technological advances, such as big data, augmented reality (AR), artificial intelligence (AI), and the Internet of Things (IoT), are changing the privacy context at an increasingly rapid pace [2-6]; devices such as Alexa collect rich data by continuously monitoring, tracking, and analyzing user behavior, which has resulted in increased concerns about their pervasiveness and user privacy [7,8]. In the context of digital health technologies, inadequate privacy policies, such as unclear or inaccessible terms, have hindered user trust and adoption, as users question the transparency and commitment of developers to safeguarding their data [9]. Broader concerns about data privacy and trust, as highlighted by incidents such as Cambridge Analytica’s exploitation of personal data and the NHS Royal Free Trust’s data sharing with DeepMind, underscore the critical need for stronger privacy governance frameworks [10].

In this study, we focused on the privacy paradox [11,12]. More specifically, although scholars have theorized that users engage in a risk-benefit assessment to weigh the benefits of sharing their data against the potential risks [13,14], numerous questions remain about the privacy paradox. For example, why are users more concerned about privacy in some contexts than others? In today’s environment, where more cutting-edge technologies are available to users, are privacy concerns changing compared to before, when they had access to just a few connected technologies, such as e-commerce websites and social media platforms? In addition, research has tended to offer a fragmented view of this phenomenon, analyzing it from several different perspectives, which points to the need to bring together and synthesize a disparate body of literature.

We posit that user privacy should be studied as a context-dependent and fluid concept rooted in the contextual integrity theory by Nissenbaum [15], because context is a critical variable in data disclosure scenarios [16-20]. Accordingly, this study aimed to make several important contributions to the field of privacy research in cutting-edge technologies.

We conducted a systematic literature review that synthesizes the various contexts in which research on privacy behaviors in IoT, AI, AR, and big data has been undertaken. The purpose of the systematic literature review was to identify the psychological antecedents, outcomes, and theories that describe privacy behavior in these new scenarios. Critically, we explore whether the findings from this body of research on cutting-edge technologies differ from previous findings in the traditional online privacy literature, such as e-commerce and social networking sites (SNS). By doing so, the study makes a valuable contribution by providing insights into how technological advancements are changing the privacy landscape and whether established privacy theories are applicable in these new contexts.

In addition, we offer a more context-contingent lens of privacy that positions context as the key variable in understanding different user behaviors in privacy scenarios. This addresses a substantial gap in the literature, as calls for the use of such a lens have so far been neglected [21,22]. This study aimed to bridge the gap toward a more context-contingent privacy discussion by examining 179 research studies and offering several theoretical contributions. The findings confirmed that contextual factors have been largely overlooked in privacy research on cutting-edge technologies, with only a small number of studies explicitly defining items aligning with the 5 parameters of information flows by Nissenbaum [15]. Our findings suggest that the perceived dichotomy between privacy concerns and behavioral intentions is not inherently paradoxical but instead reflects a lack of comprehensive understanding in a holistic and context-sensitive framework. Also, we find that past research findings in related domains (eg, e-commerce) do undergo alterations when study designs are transferred into the context of contemporary cutting-edge technologies.

The study offers significant implications for businesses, regulators, policy makers, and researchers, underscoring the importance of adopting context-sensitive privacy practices and policies. By tailoring privacy measures to the specific contexts in which cutting-edge technologies such as IoT, AI, AR, and big data operate, stakeholders can better safeguard users’ privacy while fostering trust and responsible technology adoption. These insights are also particularly relevant for health care and technology domains, where privacy concerns are critical to user acceptance and the ethical deployment of emerging innovations [23].

### Background

The rise of the internet has amplified concerns about privacy in interactions with online services. Paradoxically, however, individuals often display minimal effort or intent to safeguard their privacy [24]. Over the years, scholars have sought to explain this apparent disconnect between privacy attitudes and behaviors, using various theoretical frameworks and conceptual approaches [14,22,25]. However, to date, a widely accepted explanation of such user behavior in online disclosure situations, or the privacy paradox, has not been formulated, despite systematic efforts [13].

Two key ideas have generated considerable interest in this field. First, prior work has argued that users perform a risk-benefit assessment before disclosing data [13,14]. In this privacy calculus, perceived benefits typically outweigh perceived risks, elucidating why individuals continue to disclose information despite concerns [26]. However, research has shown that the rationality of this calculus is frequently clouded or wholly abandoned. This is due to individuals’ limited mental processing ability, which is heavily influenced by cognitive biases, heuristics [27-30], habits [31,32], knowledge deficiency [30,33], or personality traits [34,35]. These factors may even contradict the rationalism of the privacy calculus theory as a whole [13,26,35,36].

In recent years, privacy researchers have attempted to clarify the relationship between privacy attitudes and behavior. However, the literature has produced ambiguous findings, prompting some scholars to shift their focus toward contextual explanations. One such approach suggests that privacy concerns should be contextualized by examining users’ privacy concerns, corresponding antecedents, and disclosure behavior in specific information-sharing environments [37,38]. According to the theory of contextual integrity by Nissenbaum [15], privacy should be evaluated based on whether the flow of information is appropriate in a given context [39]. When informational norms are breached, privacy is violated [40]. These norms are defined by 5 parameters, namely the type of information being shared (attribute), with which recipient (actor), regarding whose information (individual), using which device (sender), and under which specific condition (transmission principles) [39,40]. Incomplete or ambiguous observations are expected if any of these parameters are missing [40]. It could be argued that the contextualization of privacy also defines how risks and benefits are perceived in the privacy calculus and what heuristics and cognitive processes are evoked.

Since privacy behavior is a highly context-dependent phenomenon, ambiguous results in the privacy literature may be explained by the context in which users experience information-sharing situations [22,41-43]. In a study by Solove [21], this argument was taken further, suggesting that the privacy paradox may not be a paradox after all because discrepancies between human behavior and attitudes do not necessarily contradict each other. Solove [21] argued that “behavior involves choices about risk in specific contexts” and “attitudes involve people’s broader valuation of privacy, often across many contexts.” Consequently, findings attempting to predict context-dependent behavior from generalized privacy attitudes will often be ambiguous, since they fundamentally measure different things (ie, general attitudes vs specific risks and benefits in a given situation).

Numerous studies have highlighted the significance of context in relation to privacy in information sharing [16-20,44]. Previous work has shown the importance of user engagement [45-47] and noted the role of transparency and open sharing of information with users [48-55]. However, despite calls within the literature to investigate the role of context in privacy in a comprehensive and theory-grounded manner [37,42,56,57], such research has largely been ignored (the study by Yun et al [24] presents a notable exception). Developing an understanding of the phenomenon that incorporates context as a critical driver of privacy concerns is essential for researchers, policy makers, and practitioners. This approach not only helps achieve more broadly applicable and less ambiguous results in privacy research but also ensures that future research, policy making, and product design decisions are more user-centric and ethically grounded [37]. For fields such as health care and digital health technologies, where privacy is a cornerstone of trust and adoption, a context-sensitive perspective is particularly crucial. By addressing privacy concerns within specific contexts, stakeholders can better design interventions, policies, and technologies that promote secure and responsible use while enhancing user engagement and compliance.

To improve our understanding of context-contingent privacy concerns it is useful to synthesize existing research to identify overarching patterns [37]. While there have been several review studies and one meta-study on the topic [11,13,14,22,24,58-60], to our knowledge, no research has yet attempted to synthesize recent findings through a contextual lens. This study aimed to fill this gap by drawing on the theory of contextual integrity by Nissenbaum [15]. In addition, context and associated challenges are constantly evolving due to technological advancements such as IoT, AI, and big data. For instance, machine learning and data aggregation techniques can now infer and predict sensitive behavior or classifications from data types that were not previously considered sensitive [61,62]. These predictions can be made even if the individual being observed has not opted in to use a particular service or product [63]. An example of this is how AI can predict sexual orientation with up to 91% accuracy from just 5 images of a person’s face [64], which Nissenbaum [40] refers to as the *data food chain* (ie, data of higher order are inferred from lower-order data).

Recent review studies, such as the ones by Gerber et al [14] and Yun et al [24], have not focused on IoT or AI technologies. In contrast, this study aimed to shed light on potential novel privacy concerns in the context of cutting-edge technologies. Yun et al [24] demonstrated that the literature on privacy has undergone significant changes in the past 2 decades, including increased focus on contextual factors. However, with the rise of IoT, AI, AR, and big data technologies, which have gained greater attention in academia due to higher adoption rates, there is a need for an up-to-date analysis. This study is also driven by recent calls for more systematic reviews to better understand the complex and ever-changing concept of “user privacy” [14,22,65].

To address this gap in the existing literature, we conducted a systematic literature review focusing on 3 key research questions: first, in what specific contexts related to information flows have privacy concerns been studied in the age of AI, IoT, big data, and AR? Second, what psychological antecedents, outcomes, and theories explain privacy behavior in these technologies? Finally, how does privacy-related behavior in cutting-edge technologies differ from prior findings in contexts, such as websites, mobile apps, SNS, or e-commerce? By addressing these questions, this study provides a synthesized overview and a crucial first step toward a context-contingent understanding of privacy, offering actionable insights for future research.

This understanding is particularly relevant for the fields of digital health and medical informatics, where privacy concerns are often heightened due to the sensitive nature of personal data. It equips policy makers and practitioners with tools to better address privacy violations, empower users with greater control over their data (eg, privacy self-management), and promote safer technological ecosystems through design principles such as privacy by design [11,12,58,66-68]. In doing so, this study can help advance our understanding and application of technology to improve user trust and system efficacy in health care and beyond.

## Methods

### Overview

We conducted a systematic literature review to offer a transparent and comprehensive analysis of the existing literature and to develop a more context-contingent understanding of privacy concerns [69-71]. Compared to traditional reviews, systematic literature reviews are less biased, more accessible, and provide higher validity because they use rigorous, scientific, and transparent methods in line with strict guidelines, which allow for replicability of results [72-75]. Systematic literature reviews are particularly helpful to achieve knowledge synthesis and can enable a broader scope than traditional narrative reviews, which is essential when addressing the interdisciplinary research questions posed in this paper [74]. Our systematic literature review adhered to the guidelines suggested in a study by Booth et al [75] and Fisch and Block [70]. A completed PRISMA 2020 checklist is provided in Multimedia Appendix 1. To ensure replicability, we provided a detailed and transparent account of our process, which we outline in the subsequent section.

### Motivation, Scope, and Systematic Literature Review

A systematic literature search was conducted using electronic databases to minimize biases and selection errors associated with manual search queries [76]. The chosen databases, ScienceDirect (Elsevier), SAGE, and EBSCO served as the primary sources for the search, given their advanced search capabilities and extensive collections of scientific articles in pertinent fields, such as business, psychology, and IT, spanning several decades.

To further enrich the search results, Google Scholar and the reference lists of relevant articles were also used to identify potentially overlooked sources [77]. Various keyword combinations, including “AI,” “IoT,” “Big Data,” “Augmented Reality,” and “Machine Learning,” were used in the search process, as these terms often encompass overlapping concepts and applications. For instance, IoT devices can operate on AI algorithms, generating ARs while collecting vast amounts of data classified as big data [78-81].

Search queries were designed to include the selected keywords in the articles’ titles, abstracts, and listed keywords. A representative and simplified Boolean search sentence used for this purpose is presented in Textbox 1. For the complete search strategy used across databases, including all keyword categories and filters, see Multimedia Appendix 2.

Furthermore, advanced search functions, such as filtering for peer-reviewed articles only, were used to streamline the search process and enhance the quality of the results. Consequently, a total of 521 articles and conference papers were identified and retrieved from the initial search conducted on the 3 primary databases, in addition to the supplementary searches on Google Scholar and relevant reference lists.

**A representative and simplified Boolean search sentence used for the systematic review.**

(TI privacy OR “privacy paradox” OR “privacy concern”) AND (“privacy concerns” OR “privacy paradox” OR personalization paradox OR perceived vulnerability OR disclosure OR perceived control OR risk OR willingness to disclose OR user OR user OR adoption) AND (internet of things OR iot OR “smart devices” OR “connected device” OR “artificial intelligence” OR ai OR big data OR machine learning OR augmented reality OR ar OR virtual reality)

### Assessing the Existing Body of Literature

The body of literature retrieved was subsequently assessed using the Covidence (Veritas Health Innovation Ltd) tool, with explicit exclusion and inclusion criteria established to systematically evaluate the existing evidence [70]. The primary objective of this initial assessment was to refine the dataset to include only the most pertinent articles that would address the research questions. To this end, duplicates (n=53) and nonrelevant papers (n=128) were removed from the total pool of 521 abstracts. Articles were deemed nonrelevant if they were not written in English or focused on different aspects of privacy research, such as technical privacy-enhancing software or ethics, rather than examining the psychological or behavioral interrelationships. Following this initial screening, 340 studies remained and underwent a full-text review for eligibility. An additional 161 articles were excluded at this stage for various reasons: they were not peer-reviewed; not empirical (including review studies); unrelated to the research objectives; of low quality (ie, lacking clarity or transparency); previously overlooked duplicates; or their full texts were unattainable [75]. As a result, the final dataset for analysis comprised 179 studies, which served as the basis for the subsequent synthesis and evaluation.

### Analyzing and Synthesizing Findings

**Figure 1:**
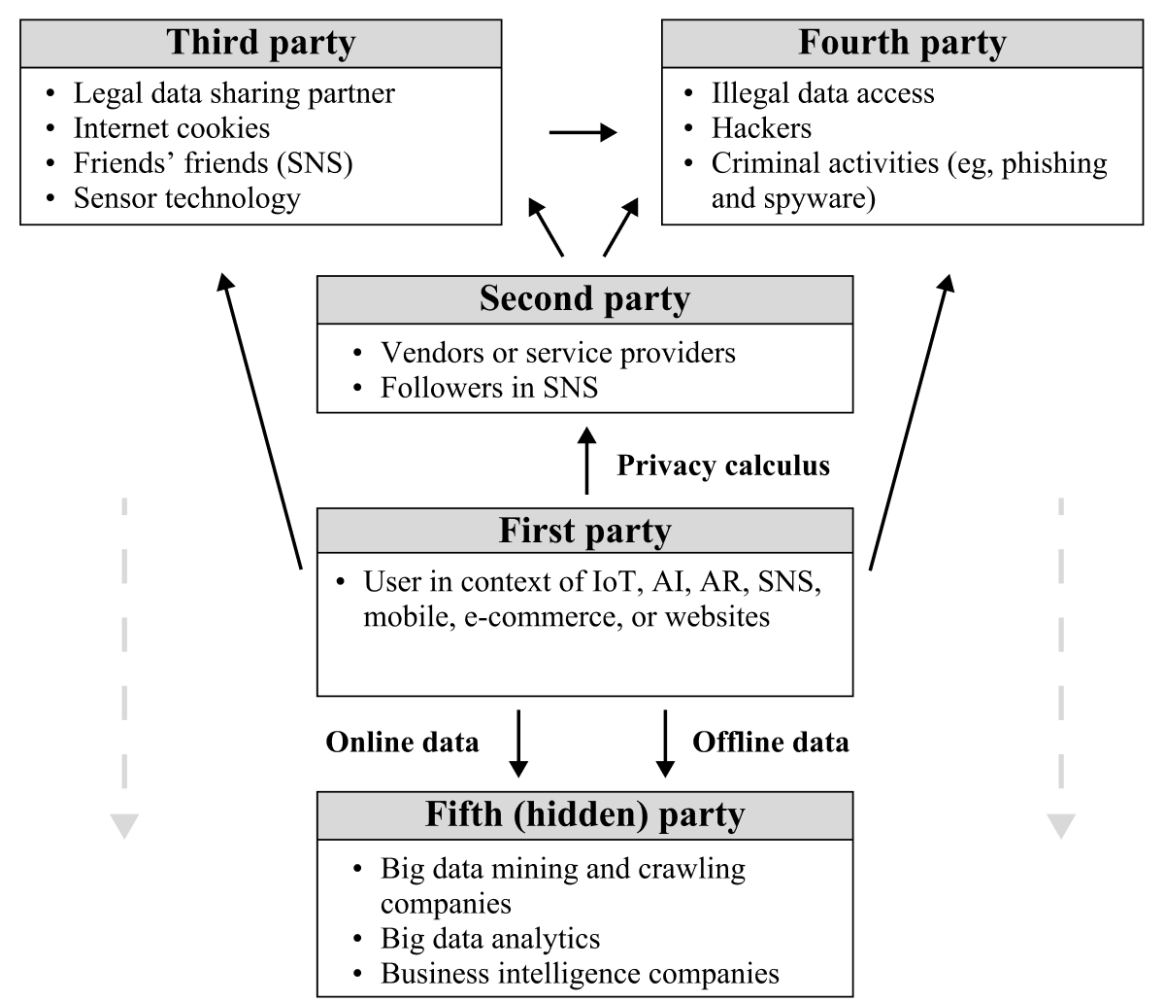
Five-party model of personal information privacy. AI: artificial intelligence; AR: augmented reality; IoT: Internet of Things; SNS: social networking sites.

In the final stage of analysis, the 179 selected studies were systematically synthesized and categorized, based on the established screening process. Following the guidelines proposed by Booth et al [75], key characteristics of each study, such as authors, title, abstract, year of publication, and the type and name of the publication, were organized in a table. Methodological factors, including study design, sample size, used theories, data collection procedures, and measures, were also documented. To address the first research question (ie, in what contexts related to information flows have privacy concerns been studied?), the analysis of the final dataset involved coding the studies according to the domains and contexts in which they were conducted. The theory of contextual integrity by Nissenbaum [15] was used, with studies coded based on the 5 parameters of information flows: data subject, sender, recipient, information type, and transmission principle. In addition, the 5-party personal information privacy model by Yun et al [24], building on the study by Conger et al [82], was adopted to gain a more nuanced understanding of the recipients of personal information (Figure 1 [24]). The model facilitated the identification of numerous, often concealed parties potentially engaged in data sharing scenarios, highlighting the complexity of privacy in advanced technologies that extend beyond a traditional sender-recipient model. Finally, the findings of each study, including graphical conceptualizations of research models if given, were incorporated into the analysis. This comprehensive approach facilitated a deeper examination of antecedents, mediators, moderators, control variables, and outcomes related to privacy attitudes and behaviors, which in turn allowed for the exploration of the second and third research objectives (ie, identifying key psychological antecedents, outcomes, and theories and comparing privacy-related behavior in cutting-edge technologies vs older technologies).

It is important to note that the codes used during this process were not predetermined; rather, they emerged inductively from the analysis of the articles [73]. Whenever a context, domain, or psychological factor (ie, antecedents or outcomes) was mentioned, a code was annotated in the full text and subsequently transferred to the table.

## Results

### Overview of Dataset

**Figure 2:**
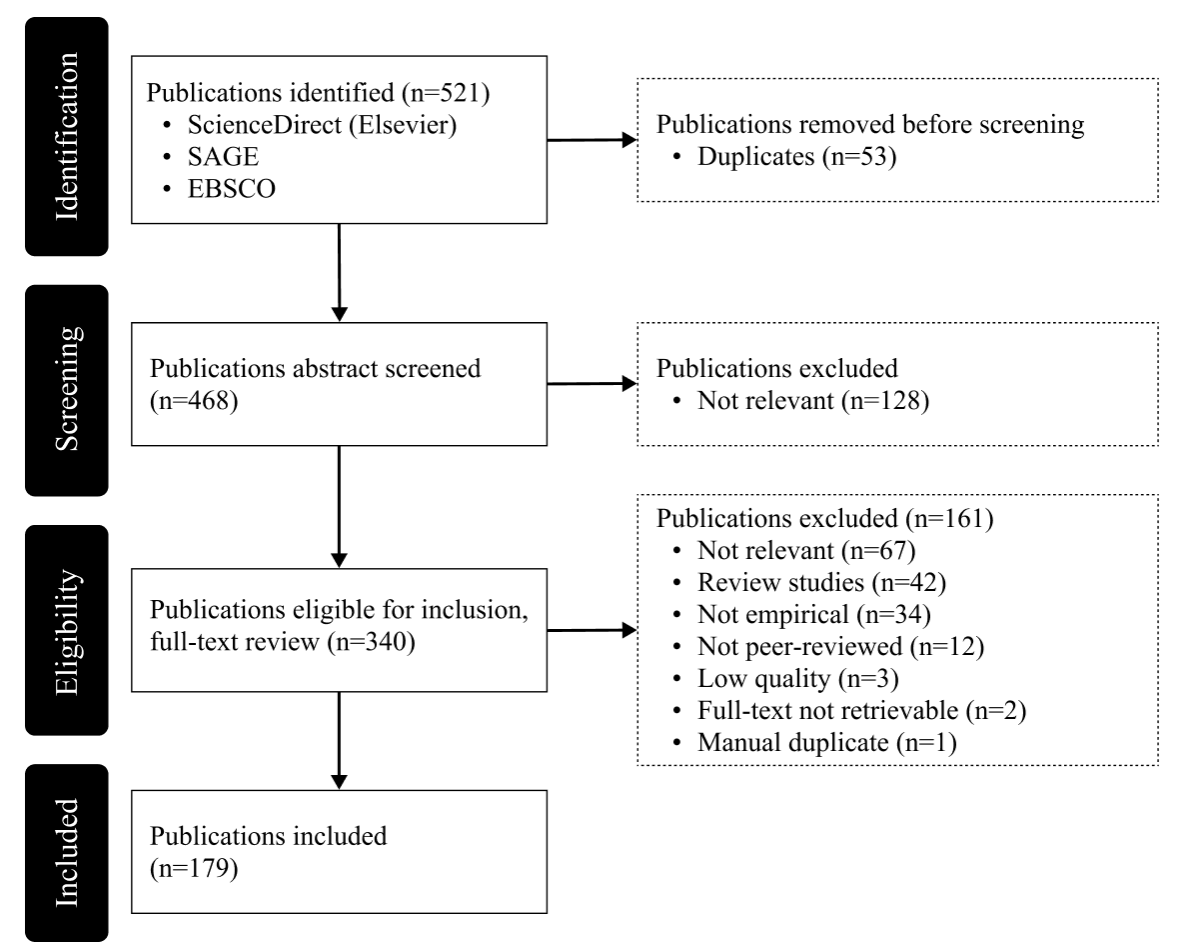
PRISMA (Preferred Reporting Items for Systematic Reviews and Meta-Analyses) flowchart.

The analysis of the final dataset comprising 179 studies aimed to provide an overview of common characteristics among the selected studies. These studies were identified through a structured screening process, as illustrated in Figure 2. The volume of relevant studies has experienced a considerable increase in recent years. The earliest article in this dataset was published in 1999, and since the early 2010s, academic interest in privacy-related topics has gained significant momentum. Notably, the number of publications doubled between 2020 and 2021, and 2022 (when this literature review was carried out) witnessed a new peak in output, as at the time of the analysis, the publication count was only 8 fewer than that in 2021. This increase aligns with the expectations, as the focus of this study is on privacy within the context of emerging technologies (ie, AI, big data, and IoT). These technologies have seen a rise in relevance and adoption rates during the same period, as can be seen in a study by Transforma Insights [5]. Moreover, the growing prominence of privacy research can be attributed to the unfolding of the information age, which has placed privacy at the forefront of academic discourse. Many scholars consider privacy to be “the issue of our times” [83], further highlighting its importance in contemporary research.

The thematic composition of journals publishing articles on privacy merits particular attention. To evaluate the quality and core focus of these journals, we used the journal quality list devised by Harzing [84], as well as the methodology outlined by Mustak et al [73]. Our analysis included 157 journal articles, 19 conference papers, and 3 book chapters. In the infrequent instances where a journal was absent from the list by Harzing [84], the authors determined the appropriate categorization by aligning the journal’s website description with a suitable classification. As there was no uniform and reliable method for categorizing conference papers and book chapters, these were grouped under “others” following the classifications proposed by Harzing [84]. Upon closer examination, it became evident that a multitude of journals from diverse knowledge areas had explored privacy as a research subject, thereby highlighting the interdisciplinary nature and relevance of the topic. However, as outlined in Table 1, journals within the information systems and knowledge management domains accounted for the largest portion of the literature, comprising 44.7% (80/179) of the total body. In addition, marketing journals made a substantial contribution (40/179, 22.4%), with nearly a quarter of the articles appearing in publications from this field.

**Table 1:** Number of publications in the various knowledge areas of journals based on the list by Harzing [84] (N=179).

Knowledge area	Publications, n (%)
Management information systems and knowledge management	80 (44.7)
Marketing	40 (22.4)
Communication	16 (8.9)
Psychology	5 (2.8)
Public sector management	4 (2.2)
Operations research, management science, and production and operations management	3 (1.7)
Tourism	3 (1.7)
Organization behavior or studies, human resource management, and industrial relations	1 (0.6)
Innovation	1 (0.6)
Economics	1 (0.6)
Others (conference paper, book chapter, or not matching the category in the list by Harzing)	25 (14)

During the analysis, each paper was assigned a code corresponding to a primary theme. This theme was predominantly determined by the device used in study designs (ie, the sender parameter) of an article. For instance, if a study examined privacy concerns among mobile app users, the “mobile” code was assigned. In cases where chatbot recommendations elicited privacy concerns, the “AI” code was used. When a specific sender was not identified, the thematic topic was recorded, and the “other” code was applied. This coding system aimed to offer a concise overview of the research focus in contemporary literature, facilitating our analysis in subsequent stages. However, it is important to acknowledge that these codes can be considered subjective and potentially ambiguous due to the often closely related or synonymous terminologies in the field. For example, a voice assistant (ie, IoT) could also be classified as AI. Despite this, the results presented in Table 2 reveal that the primary research focus areas thus far have been related to IoT, SNS, mobile, and AI. Finally, upon excluding nonempirical findings, it was observed that most studies (146/179, 81.6%) exhibited a quantitative methodological orientation, with only a small number of studies (17/179, 9.5%) adopting purely qualitative methods or implementing a mixed methods approach (16/179, 8.9%).

**Table 2:** Number of predominant themes that emerged from the analyzed studies (N=179).

Predominant theme			Publications, n (%)
Internet of Things			48 (26.8)
Social networking sites (including social media and instant messengers on mobile or websites)			29 (16.2)
Mobile (including apps, location-based services, and mobile e-commerce)			20 (11.2)
Artificial intelligence			20 (11.2)
Big data			9 (5)
Website (including e-commerce)			9 (5)
Augmented reality			7 (3.9)
**Other (unspecified)**			37 (20.7)
	In e-commerce	7 (3.9)	
	General online privacy concerns	30 (16.8)	

### Analysis of Context

#### Overview

Table 3 shows the results of the analysis of contexts (ie, information flows) used to study privacy concerns in the age of cutting-edge technologies (ie, AI, IoT, big data, and AR). Results for each contextual dimension are discussed subsequently.

**Table 3:** Number of contextual dimensions (ie, information flow parameters) of analyzed studies.

Contextual dimension and code			Publications, n (%)
**Subject** **(n=179)**			
	User (IoT^a^)	50 (27.9)	
	User (online)	37 (20.7)	
	User (SNS^b^)	30 (16.8)	
	User (AI^c^)	18 (10.1)	
	User (mobile)	18 (10.1)	
	Shopper (e-commerce)	13 (7.3)	
	User (AR^d^)	7 (3.9)	
	Citizen	2 (1.1)	
	User (big data)	2 (1.1)	
	Employee	2 (1.1)	
**Sender** **(n=179)**			
	IoT	51 (27.9)	
	SNS	28 (15.6)	
	Website	26 (14.5)	
	Mobile	24 (13.4)	
	AI	18 (10.1)	
	Online or offline general total	23 (12.8)	
	Multiple sender	5 (2.8)	
**Recipient (actor;** **n=179)**			
	Analyzed with varying sensitivity	26 (14.5)	
	Not analyzed for sensitivity	123 (68.7)	
	Not specified	30 (16.8)	
**Type of information (attribute; n=184)^e^**			
	General and unspecified personal information	74 (41.3)	
	Specified personal information total	*97* (54.2)^e^	
	Not specified (ie, not mentioned)	13 (7.3)	
**Transmission principle (n=192)^e^**			
	Specified	50 (*28.4*)^e^	
	Not specified	142 (79.3)	

^a^IoT: Internet of Things.

^b^SNS: social networking sites.

^c^AI: artificial intelligence.

^d^AR: augmented reality.

^e^Number and percentage in the table indicate how often a parameter was found in a paper. The same paper may belong to multiple categories in a given dimension. The percentage was calculated with a base value of 179. For example, in 79.3% of papers, no transmission principle was specified.

#### Individuals (Subjects) Whose Data Are Being Collected

Data collection subjects were categorized into 10 groups based on how articles delineated their respondents within study designs. For instance, a user could be perceived as a generic online user [85], an IoT device user [61], or a citizen interacting with an e-government platform [86,87]. A substantial majority of studies closely adhere to the device used in study designs (ie, the sender) and implicitly or explicitly define users accordingly. Exceptions included extracted codes for designs specifically focusing on employees (2/179, 1.1%), e-commerce shoppers (13/179, 7.3%), and citizens (2/179, 1.1%). The 3 most prominent types were IoT users (50/179, 27.9%), generic online users (37/179, 20.7%), and SNS users (30/179, 16.8%). These results largely align with our expectations.

However, the low counts for AR users were unexpected, given that the terms “augmented” and “virtual reality” were part of the initial search query. The impressive market growth rates and relevance across multiple sectors also suggested higher academic significance [6,88]. Moreover, it is worth noting that, despite our up-to-date dataset, SNS continue to attract considerable attention, even though they were not included in our initial search query [89-92]. To maintain manageable results, we opted against coding with even greater granularity. For example, a few studies concentrated on specific demographic groups such as children [93,94], teenagers [95], or individuals with low socioeconomic status [96]. Most studies in our dataset controlled for demographics and incorporated corresponding variables, such as age or gender.

#### Devices (Sender) Used to Transmit Personal Information

The sender parameter results offer a detailed perspective on the specific technologies examined by studies in our dataset. In accordance with findings in the user category, we observe that IoT senders represent the most substantial share in this dimension (51/179, 27.9%). However, when using a more intricate coding, we notice differences in the particular device types. For example, the most prominent single sender type is not an IoT device but rather SNS (28/179, 15.6%), followed by websites (26/179, 14.5%) and mobile devices (24/179, 13.4%). IoT smart assistants ranked as the fourth most prevalent device type within the dataset (21/179, 11.7%). The high occurrence of SNS senders in the sender parameter was unexpected, prompting further investigation. Most of the analyzed SNS studies in our contemporary sample focused on Facebook users [89-92]. Despite their popularity and growth rates, Instagram and TikTok played no role in the SNS studies within our dataset.

Notably, only 2.8% (5/179) of studies investigated 2 different sender types concurrently, and merely one of them incorporated 4 distinct devices in its study design. This is despite recent evidence suggesting that user devices can impact privacy concerns, such as smartphones increasing privacy disclosure intentions compared to PCs [97,98]. In addition, 12.8% (23/179) papers did not explicitly define a sender and offered a more general level of analysis. Upon closer examination, we found that the absence of a sender type definition was frequently associated with a relatively early publication year [99-101]. Another reason was the investigation of another contextual dimension, necessitating control for other dimensions such as information sensitivity [18,102,103]. Over recent years, the privacy literature has arguably developed a more nuanced understanding concerning the definition of the sender dimension in studies [24].

#### Recipient of Personal Information

A particularly intriguing contextual dimension concerning cutting-edge technologies pertains to the recipient of personal information, as the methods of data collection and sharing with second, third, fourth, or even fifth parties are rapidly evolving (Figure 1) [24]. The concept previously referred to as the “data food chain” [40] highlights the significance and complexity of incorporating this dimension into study designs. Through big data mining, data aggregation, and data analytics, organizations can now predict online and offline behavior using vast, seemingly unrelated data points [24,61,62,104].

However, only Hermes et al [105] and Xie and Karan [92] have explicitly examined the so-called hidden fifth party (2/179, 1.1%). Most articles (146/179, 81.6%) primarily focus on the direct transaction between a user (first party) and the manufacturer or provider of a device or service (second party). Contrary to findings in Yun et al [24], the intended or unintended sharing with third parties was not as prevalently studied in our dataset as anticipated (22/179, 12.2%). Nevertheless, we observed a similar trend to the cited study, with more recent articles beginning to investigate illegal, unauthorized secondary access by fourth-party entities (9/179, 5%). Moreover, a mere 14.5% (26/179) papers in the sample considered varying the sensitivity of the data recipient while examining privacy concerns, such as altering the sharing of personal information with device manufacturers or third-party advertisers. Some notable examples include studies by Abdi et al [61], Lee and Kobsa [106], Leom et al [39], Lutz and Newlands [107], Marmion et al [108], and Martin and Nissenbaum [19]. However, almost 70% (123/179) of the analyzed articles did not investigate users’ varying perceived sensitivity toward recipients of their personal information, despite the increasing importance of this dimension, particularly in the IoT, AI, AR, and big data era [24]. The implications of this will be discussed later in the paper.

#### Type of Information Being Shared

The idea that variations in privacy behavior may also depend on the type of information shared with an entity was introduced relatively early in the literature [109,110]. Our analysis revealed that more than half of the studies (97/179, 54%) mention a specific type of information being collected in their experiments or designs. Several studies in this set either focus on a particular information type exclusively (37/179, 20.7%) or investigate multiple information types without further categorization (ie, examining credit card details and purchase history, among other things, without classifying them as “financial” or “transactional” information; 22/179, 12.3%). Although a significant number of articles do not specify the type of personal information collected (74/179, 41.3%), we observed a trend indicating that an increasing number of researchers are varying the sensitivity of information types to obtain more meaningful and comprehensive results (38/179, 21.2%). In fact, 24 (13%) out of the 38 studies that experiment with variation in this parameter were published within the last 5 years. Notable examples among these include studies by Abdi et al [61], Apthorpe et al [17], Carignani and Gemmo [111], Cichy et al [112], Leom et al [39], Markos et al [18], Markos et al [113], and Marmion et al [108].

#### Transmission Principles in Data Sharing Situations

The principles governing data transmission relate to the why and under what conditions personal information is being shared with recipients. For instance, Abdi et al [61] discovered that the acceptability of data sharing scenarios is contingent upon factors, such as the retention duration, user notification regarding data usage, and assurances of anonymity and confidentiality. Lee and Kobsa [106,114] corroborate these findings, highlighting the significance of the rationale behind personal data collection in shaping users’ judgments. For instance, users may perceive data collection for safety-related purposes as less acceptable than for health-related purposes [114]. Moreover, IoT devices, such as smart home assistants, are constantly monitoring for voice commands (eg, “Hey Google,” “Siri”), prompting a limited number of studies (4/179, 2.2%) to explore potential differences in user reactions to continuous versus onetime monitoring [115]. The context in which data are transferred, specifically public versus private settings, also influences privacy concern assessments [43,98,116]. A significant aspect of data transmission principles, particularly in the context of advanced technologies, pertains to the mode of data collection—whether it occurs covertly or overtly. This distinction hinges on whether users are aware of or can observe the data collection process [89,117-119].

Despite existing evidence that these data transmission principles influence participants’ judgments in data sharing situations, the literature on this subject remains limited. A review of empirical studies in the field reveals that 79.3% (142/179) do not explicitly address this contextual dimension. Most of these studies tend to implicitly explore certain principles by examining a specific service, such as Facebook, whose privacy policy articulates some transmission principles. However, only a handful of studies incorporate reading privacy policies or cookie notifications into their research design [120]. Consequently, future research should more explicitly incorporate and investigate data transmission principles as a parameter [98].

### Antecedents and Outcomes

#### Overview

**Figure 3:**
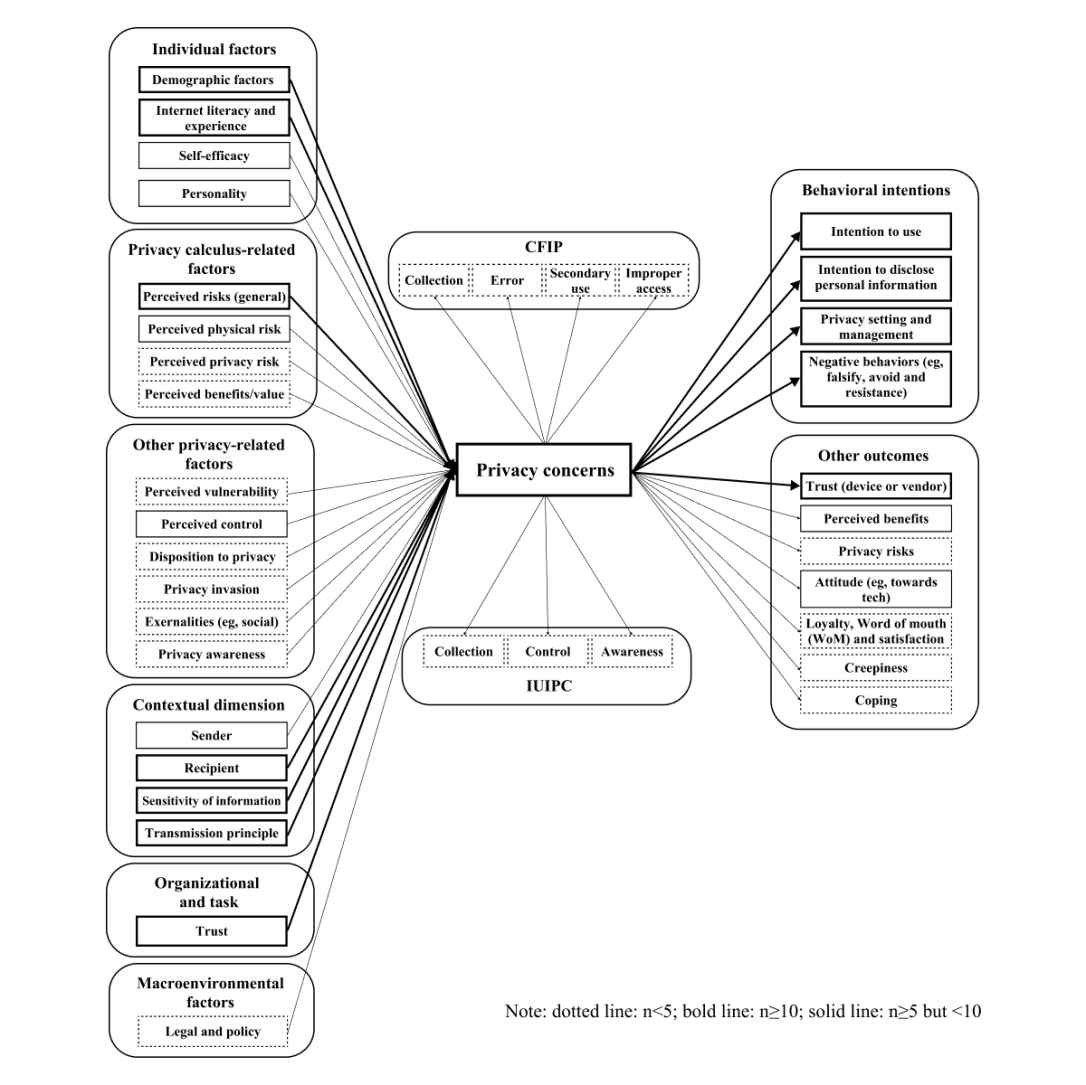
Count of antecedents and outcomes of privacy concerns in the context of Internet of Things (IoT), artificial intelligence (AI), augmented reality (AR), and big data. CFIP: concerns for information privacy; IUIPC: internet users’ information privacy concerns; WoM: word of mouth.

We analyzed a subset of our sample more closely to investigate which antecedents, outcomes, and theories were considered in the literature on cutting-edge technologies, thus addressing our second main research objective. We extracted studies coded with a predominant theme of IoT, AI, AR, or big data (Table 2). Our subset of 68 studies is mainly composed of articles that were published no later than 2017. We synthesized findings and subsequently categorized study results into antecedents, privacy concerns, and outcomes (Figure 3). Some constructs such as trust, perceived risks, or perceived benefits were found to act as antecedents or outcomes.

Building upon and extending the work of Yun et al [24], we categorized antecedents into 6 distinct classifications: individual factors; privacy calculus-related elements (ie, the perceived risks and benefits users evaluate in their risk-benefit analysis before data disclosure); additional privacy-related aspects; organizational and task-related factors; macroenvironmental influences; and contextual variables [121]. Our integrative and succinct model uncovers previously unexplored findings from privacy research in the context of emerging technologies. For example, we observe the emergence of novel antecedents such as perceived physical risks (eg, users’ apprehensions regarding physical harm or intrusion into their personal space), which have been largely overlooked in past research [121]. As technological advancements progress, so do the contexts and human behaviors in which they are embedded. The notion of physical risk exemplifies this interrelationship, as it is intimately connected to the unique features of IoT, AR, and AI devices [122]. In this regard, Cichy et al [112] demonstrated that drivers of connected and autonomously driven vehicles are increasingly anxious about their physical well-being, as concerns about the safety of autonomous driving are evoked. Similarly, Pal et al [121] underscore the significance of physical risks for users of smart home devices. The integration of these novel IoT technologies within a user’s personal space generates apprehension about surveillance and fosters fear of losing control over one’s private environment [121].

In examining the outcomes of privacy disclosure within the realm of emerging technologies, our analysis categorizes findings into 2 groups: behavioral intentions and other outcomes [24]. Several novel constructs have surfaced in recent years that were previously unaddressed [24], and these are intrinsically connected to the unique attributes of cutting-edge technologies. For instance, Rajaobelina et al [123] demonstrated how privacy concerns can heighten users’ sense of unease or “creepiness” during interactions with AI-based chatbots. Marakhimov and Joo [124] presented empirical evidence indicating that user privacy concerns related to wearable health devices prompt coping mechanisms (ie, attempts to reinterpret or alleviate negative emotions), potentially explaining the abandonment of wearables by users within months of acquisition [125]. Moreover, our analysis revealed an increasing number of studies that explore a wider array of outcome variables, extending beyond direct links to use intentions. For example, Cheng and Jiang [126] discovered that perceived privacy risks associated with chatbots can lead to diminished satisfaction, while Rajaobelina et al [123] highlighted the influence of privacy concerns on customer loyalty, mediated by factors such as creepiness, trust, and negative emotions.

Taken together, we discern several notable patterns within our subset of recent articles focusing on emerging technologies. First, a significant number of studies investigated personality traits as antecedents [34,127]. The examination of personality factors as antecedents of privacy concerns is a relatively nascent area of focus, initially identified by Yun et al [24], which has since proliferated throughout the broader literature.

Second, numerous contextual factors have been incorporated into examinations of privacy concerns as they relate to IoT, AI, AR, or big data technologies. Notably, sensitivity of information emerged as a frequently incorporated antecedent within our 68-study subset. This finding aligns with previous results in the overall sample (Table 3). Contextual dimensions have gained prominence in contemporary empirical inquiries of privacy concerns in the context of cutting-edge technologies.

Third, it is noteworthy that none of the papers in our subset explicitly explore cultural values. While sample recruitment spanned countries such as Jordan [128], Saudi Arabia [129], Turkey [130], India [131], and China [132], no article in our subset examined privacy concerns in the context of IoT, AR, AI, or big data across national boundaries. However, such studies are present in our complete dataset of 179 articles when the focus on cutting-edge technology is removed. For instance, Pentina et al [133] conducted a cross-cultural comparison in the context of mobile app adoption, while Markos et al [113] explored differences in perceived sensitivity and willingness to disclose between US and Brazilian online users. Finally, we observe that trust as an antecedent and the outcome construct of behavioral intention are the most prevalent themes within the analyzed subset, yielding most results. This propensity has persisted in the privacy literature, as demonstrated by the chronological review of Yun et al [24] encompassing a sample of studies from 1996 to 2017.

#### Theories in the Context of IoT, AI, AR, and Big Data

In our investigation, we also examined the frameworks and theories used to study IoT, AI, AR, and big data technologies. Drawing on the study by Barth and de Jong [13], we classified theories into 3 categories. The first category encompassed theories predicated on the assumption that users’ underlying risk-benefit evaluations are primarily rational. The second category comprised theories positing that rational privacy valuations are occasionally influenced by irrational biases (eg, heuristics, immediate gratifications, and habits). Finally, the third category, labeled “other” was designated for theories adopting alternative approaches to explain behavior in information-sharing situations. Table 4 provides a summary of the theories identified in our 68-study subsample.

**Table 4:** Overview and categorization of theories in analyzed subset (IoT, AI, AR and big data).

Category and theory		Studies	Exemplary studies
**Risk-benefit calculation guided by rationality**			
	Justice theory	Deutsch [134]	Pizzi and Scarpi [135]
	Multidimensional development theory	Laufer and Wolfe [136]	Pal et al [121]
	Privacy calculus	Culnan and Armstrong [100]	Cheng et al [2]; Hermes et al [105]; Liu et al [132]; Attié and Meyer-Waarden [137]; Gao et al [138]; Kang and Jung [139]; Kim et al [140]; Lavado-Nalvaiz et al [141]; Willems et al [142]
	Protection motivation theory	Rogers [143]	Gao et al [138]; Alashoor et al [144]; Thongmak [145]; Williams et al [146]
	Social exchange theory	Blau [147]; Homans [148]	Uysal et al [149]
	Technology acceptance model	Davis [150]	Yavuz et al [130]; Pizzi and Scarpi [135]; Attié and Meyer-Waarden [137]; Liu and Tao [151]; Park et al [152]
	Theory of planned behavior	Ajzen [153]	Hermes et al [105]; Attié and Meyer-Waarden [137]; Alashoor et al [144]
	Theory of reasoned action	Ajzen and Fishbein [154]; Fishbein and Ajzen [155]	Attié and Meyer-Waarden [137]; Bawack et al [34]
	Unified theory of acceptance and use of technology	Venkatesh et al [156]	Easwara Moorthy and Vu [116]; Attié and Meyer-Waarden [137]; Gao et al [138]; Vimalkumar et al [157]
	Communication privacy management or information boundary theory	Petronio [158,159]	Kang and Jung [139]; Cao and Wang [160]; Kathuria and Kathuria [161]
**Biased risk assessment within the risk-benefit calculation**			
	Contextual integrity	Nissenbaum [15]	Apthorpe et al [17]; Abdi et al [61]; Agesilaou and Kyza [93]; Lutz and Newlands [107]; Harborth and Pape [162];
	Coping theory	Lazarus and Folkman [163]	Cheng et al [2]; Marakhimov and Joo [124]
	Heuristic-systematic model of information processing	Chaiken [164]	Shin [165]
	Innovation resistance theory	Ram [166,167]	Pal et al [121]; Liu et al [132]
	Prospect theory	Kahneman and Tversky [168,169]	Jain et al [170]
	Signaling theory	Spence [171]	Jain et al [170]
	Social cognition theory	Bandura [172]	Cao and Wang [160]
	Uncanny valley theory	Mori [173]; Mori et al [174]	Lavado-Nalvaiz et al [141]; Willems et al [142]
	Uses and gratification theory	Katz and Blumler [175]; Katz et al [176]	Attié and Meyer-Waarden [137]; Jain et al [170]; McLean and Osei-Frimpong [177]; Rauschnabel et al [178]
**Others**			
	Mind perception theory	Gray et al [179]	Uysal et al [149]
	Query theory	Johnson et al [180]	Sun et al [181]
	Theory of diffusion of innovation	Rogers [182]; Rogers et al [183]	Attié and Meyer-Waarden [137]

Our findings indicated that most theories in our subsample were also recognized by Barth and de Jong [13]. However, formerly prominent theories such as the theory of incomplete information [184] and the theory of bounded rationality [185] were not detected in our analysis, despite using a broader search scope than Barth and de Jong [13]. Conversely, new theories have emerged, including the uncanny valley theory [173,174] and the theory of contextual integrity [15]. The former is explicitly connected to the perceived anthropomorphism of robots (ie, the degree of human-likeness) and thus aligns with our emphasis on cutting-edge technologies. This theory appeared to be the most prevalent framework in studies investigating the context-dependency of privacy disclosure, a research focus that has gained traction in recent years [17,61,107,162].

In sum, we observed an equitable distribution between theories focused on rational risk-benefit calculations and those addressing biased evaluations due to human cognitive limitations and exploited heuristics (Table 4). Within the rationality-based school of thought, the privacy calculus theory [100] and technology acceptance models, such as the Technology Acceptance Model by Davis [150] or Unified Theory of Acceptance and Use of Technology by Venkatesh et al [156], were most frequently used as the theoretical frameworks in our subsample. Regarding the second group, we found that Petronio’s [158] Communication Privacy Management and the previously mentioned theory of contextual integrity [15] emerge as the most prominent within their category.

#### Privacy Behavior in the Context of IoT, AI, AR, and Big Data

In the final stage of our investigation, we wanted to elucidate whether privacy-oriented behaviors, such as the privacy paradox, in emergent technologies tend to diverge from previous findings in other technological domains, including websites, mobile apps, SNS, and e-commerce. Most extant studies corroborated the existence of the privacy paradox. For example, Lavado-Nalvaiz et al [141] and Lutz and Tamó-Larrieux [186] highlighted the pervasiveness of the paradox within the realm of social robots. Similarly, Willems et al [142] substantiated its presence in the context of citizens’ adoption intentions concerning AI-based public service applications. Furthermore, Chaparro Osman et al [187], Aleisa et al [129], Jaspers and Pearson [188], and Pal et al [189] provided support for the privacy paradox among users of domestic IoT devices. Thus, the literature affirms the persistence of the privacy paradox across various technological contexts.

However, a more comprehensive examination of our analysis unveils intriguing findings in the literature on state-of-the-art technologies. For instance, Menard and Bott [190] discovered that privacy concerns for IoT users are addressed differently compared to those in e-commerce settings. In the latter, users typically react to heightened awareness of data collection with increased privacy concerns and consequently, protective behavior. In contrast, the authors revealed an inverse relationship in an IoT context, where awareness of data collection by the IoT device leads to greater disclosure intentions [190]. They postulate that this relationship may arise because a user’s information disclosure is intrinsically linked to the utility of IoT devices, which provide optimized and automated recommendations [190]. IoT devices possess minimal functionality unless users supply the requisite personal information, an assertion corroborated by Pal et al [189].

In a similar vein, Sun et al [181] demonstrated in an IoT experiment—comparing participants’ actual behavior with stated intentions—that participants disclosed less personal information than they initially claimed they would. The privacy paradox typically implies an intention-behavior gap in the opposite direction (ie, individuals divulging more information in behavioral conditions than intention conditions). The authors proposed that the reversed intention-behavior gap they observed could be attributed to the abstract nature of IoT devices, rendering the concept of IoT less salient than, for example, commerce [181]. Another plausible explanation for the reversed intention-behavior gap lies in users’ unfamiliarity with novel IoT technologies, leading to inaccurate risk and benefit assessments due to a lack of knowledge [181,191]. Comparable effects were demonstrated by Chaparro Osman et al [187], Hermes et al [105], and Alashoor et al [144] in IoT and big data contexts, respectively. Therefore, further research to examine the aforementioned claims is highly deserving.

## Discussion

### Contributions to Theory and Directions for Future Research

Privacy has emerged as one of the most pressing concerns of our era and will require heightened attention with the mainstream adoption of cutting-edge technologies, including IoT, AI, AR, and big data. Despite more than 2 decades of scholarly research on privacy, a comprehensive and context-dependent theory remains elusive. Consequently, a research gap persists regarding the differing standards individuals apply to data protection across various contexts [32,79]. The present systematic literature review, encompassing 179 research studies, offers several theoretical contributions in response.

By examining state-of-the-art technologies, we present a timely and distinctive perspective on how constructs and theories have evolved and been contextualized within existing privacy research. Our synthesis of findings contributes to the literature by unveiling gaps and unanticipated relationships between privacy concerns and behaviors in the era of ubiquitous computing, which could potentially guide future research. This study substantiates the notion that contextual factors have been largely overlooked in privacy research on cutting-edge technologies (our first main research question). Merely 5 studies in our dataset explicitly defined items aligning with most of the 5 parameters of information flows described in a study by Nissenbaum [15]. An analysis of perceived information sensitivity degrees was most prevalent in studies using contextualization (97/179, 54.2%). These results may support the assertions made in studies by Solove [21] and Kokolakis [22] that the dichotomy between privacy concerns and behavioral intentions is not paradoxical but, rather, has not been comprehensively understood in a holistic and context-contingent manner, which could provide logical explanations for this seemingly inconsistent behavior, also described in studies by Martin and Murphy [59]. Following Xu and Zhang [37], our findings, therefore, also suggest that if no context-contingent theory is provided and widely accepted, future researchers must use careful theory-driven contextualization to achieve more generalizability and interpretability of findings.

Future research may thus focus on examining additional information-sharing situations with emerging technologies to delineate context-dependency in privacy behavior. Specifically, additional research is needed to explore the role of institutional trust in shaping privacy concerns, as patient perceptions of trusted organizations such as national health institutions versus commercial entities significantly influence their willingness to share sensitive data [23]. This is particularly important because these technologies often operate ubiquitously without an individual’s awareness, becoming increasingly pertinent in numerous aspects of daily life, such as health care and e-government.

As recommended in the study by Yun et al [24], future studies on privacy concerns could also concentrate on these underexplored fields of application or investigate how the ubiquity and unique attributes of novel technologies alter user privacy behaviors. Our analysis demonstrated that results are frequently connected to users who are not yet familiar with emerging technologies. We encourage future research to examine how these findings evolve as new technologies become more widely adopted and users inevitably become more accustomed to these devices in their daily lives. In addition, we advocate for more studies to incorporate a broader array of specified principles in research designs, as well as examine actual behavior rather than intentions. Both aspects have been largely neglected in the literature, yet they significantly influence study outcomes [192].

Our findings further indicate that most antecedent and outcome constructs, along with underlying theories, have been transferred from research on websites, social media, or e-commerce to the context of cutting-edge technologies (our second key research question). We observe that the literature continues to develop or use novel constructs and theories frequently associated with unique characteristics of IoT, AR, or AI devices (eg, physical risks). While the ongoing expansion of constructs may be necessary to comprehend new technologies, it simultaneously introduces further divergence in a field already replete with theoretical approaches [24].

Our study supports the call for developing context-contingent insights specific to emerging technologies and work that enhances user engagement and well-being [22,24,193-202], as well as converging toward more generalizable, robust, and underlying theories that systematically operate across diverse contexts [24,37]. The limited privacy literature on cutting-edge technologies reveals that previously identified results in other domains, such as e-commerce, do undergo alterations when study designs are recontextualized into IoT, AR, AI, or big data (our third key research question). More context-specific studies on privacy concerns in cutting-edge technology scenarios are needed to reach a critical mass to allow a theoretical synthesis of findings [37]. As previously mentioned, these studies must be theory-grounded to allow generalizability. We argue that using the 5 parameters of the contextual integrity theory described in a study by Nissenbaum [15] in future research will allow a synthesis toward a context-contingent understanding of privacy. We posit that individuals’ privacy concerns and behaviors are dependent upon a complex interplay of contextual dimensions inherent to IoT, AI, AR, and big data technologies rather than a rational risk-benefit analysis. Specifically, these dimensions include the individuals whose data are being collected, the devices transmitting personal information, the recipient of the information, the type of information shared, and the principles governing data transmission. We thus suggest that the dynamics within contextual dimensions of emerging technologies contribute to shaping individuals’ privacy attitudes, intentions, and behaviors, leading to unique patterns that may differ from traditional contexts.

### Implications

First, the results underscore the critical role of contextual dimensions in shaping user privacy concerns, emphasizing the need for companies and device manufacturers to investigate these dimensions thoroughly. Context-aware privacy considerations can inform the design and marketing of new digital health technologies, enabling products that align with user preferences and ethical standards. For example, the development of transparent and accessible privacy policies tailored to user needs can address significant user concerns, as demonstrated in previous studies on the impact of clear privacy policies on user trust and adoption [9,10]. In addition, the use of conversational AI in health care has been shown to mitigate privacy concerns by streamlining user engagement while ensuring data security, as evidenced by recent implementations [203].

Adopting a “privacy by design” approach might ensure that products, such as wearable health devices or telehealth platforms, offer granular control over sensitive data without overwhelming users with excessive opt-in choices [98]. Overloading users with consent requests may lead to decision fatigue, reducing their sense of control and trust in the system [204]. Instead, default privacy-preserving options can effectively guide user behavior while minimizing intrusiveness, particularly for less sensitive data streams [205]. Health care–focused applications of IoT and AI, such as remote monitoring devices, could integrate these insights to enhance usability and compliance while safeguarding patient data. For example, enabling users to manage privacy settings for specific contexts, such as sharing data with health care providers but not with third-party advertisers, can improve trust and engagement with these technologies.

Marketing and customer service teams in the digital health space can benefit from a deeper understanding of user privacy concerns in specific scenarios. By acknowledging the perceived risks associated with IoT devices, especially in health care (eg, fears of data breaches or misuse of sensitive health data), companies can develop targeted educational campaigns to address these concerns. Clear, transparent communication about data use, security protocols, and user controls can alleviate anxiety, fostering higher levels of trust, satisfaction, and loyalty. Enhanced trust in digital health platforms can translate into increased adoption rates and long-term engagement, ultimately benefiting both users and organizations [206,207].

From a policy-making perspective, our findings highlight the need to revise and refine privacy regulations to address the contextual nuances of privacy behavior. Policy makers should mandate that privacy policies explicitly outline all 5 contextual parameters proposed by Nissenbaum [15,38], ensuring that users understand how their data are collected, shared, and used in specific contexts. This is particularly important for digital health technologies, where sensitive health information often flows through complex data ecosystems, involving multiple parties. Transparent policies that restrict information sharing to essential second- or third-party recipients can reduce unauthorized data flows and prevent misuse [17].

Moreover, regulators should establish standards for “privacy by design” in the development of new health technologies, requiring restrictive default settings and clear consent mechanisms. For example, IoT-enabled medical devices could be required to limit data sharing to approved health care providers, reducing the risk of exposure to unintended parties. In addition, ethical guidelines for predictive analytics, such as algorithms that infer health conditions from user behavior, should ensure that these insights are used responsibly and transparently, avoiding harm to users or erosion of trust.

### Conclusions

This study underscores the critical importance of adopting a context-sensitive lens to understand privacy concerns in the rapidly evolving landscape of emerging. By synthesizing existing studies, this work uncovers significant research gaps and emphasizes the need for more holistic, context-sensitive approaches that consider the dynamic interaction between psychological antecedents, behavioral outcomes, and technological attributes. Our findings have important implications for researchers, practitioners, and policy makers alike. For researchers, they provide a foundation for advancing theories that are adaptable to diverse and complex privacy scenarios. For practitioners and technology developers, the insights offer a pathway to designing user-centric technologies that integrate privacy as a core feature rather than an afterthought. For policy makers, this work emphasizes the necessity of revising privacy frameworks to address the contextual dimensions of data sharing and ensure ethical and transparent practices. In domains such as digital health, where the stakes are particularly high, such an approach can build trust, enhance user engagement, and support the ethical deployment of technologies that improve health outcomes and empower individuals. By bridging gaps in the literature and paving the way for context-driven solutions, this study contributes to the broader goal of fostering a digital ecosystem that balances innovation with user privacy and trust.

## Data Availability

The datasets generated or analyzed during this study are available from the corresponding author on reasonable request.

## Appendix

Multimedia Appendix 1 PRISMA (Preferred Reporting Items for Systematic Reviews and Meta-Analyses) checklist.

Multimedia Appendix 2 Outline of Search Strategy.

## Abbreviations

AI: artificial intelligence
AR: augmented reality
IoT: Internet of Things
SNS: social networking sites
