# Supplementary material for: Context-Contingent Privacy Concerns and Exploration of the Privacy Paradox in the Age of AI, Augmented Reality, Big Data, and the Internet of Things: Systematic Review
Source: J Med Internet Res. 2025 May 14;27:e71951. doi: 10.2196/71951 (PMC12120372; doi:10.2196/71951)
Supplement: Multimedia Appendix 2 [file jmir_v27i1e71951_app2.docx]

**Multimedia Appendix 2**

**Primary sources:** ScienceDirect (Elsevier), SAGE, and EBSCO

**Keywords:**
Table S1: Complete list of all terms and keywords used in search strategy.

| **Category** | **No.** | **Keywords** |
| --- | --- | --- |
| **Category 1 (Title)** | 1 | Privacy |
|  | 2 | Privacy Paradox |
|  | 3 | Privacy Concern |
|  | 4 | Privacy Concerns |
| **Category 2** | 5 | Privacy Concern |
|  | 6 | Privacy Concerns |
|  | 7 | Privacy Paradox |
|  | 8 | Personalization Paradox |
|  | 9 | Perceived Vulnerability |
|  | 10 | Disclosure |
|  | 11 | Consumer |
|  | 12 | User |
|  | 13 | Adoption |
| **Category 3** | 14 | Internet of Things |
|  | 15 | IoT |
|  | 16 | Smart Device |
|  | 17 | Smart Devices |
|  | 18 | Connected Device |
|  | 19 | Connected Devices |
|  | 20 | Artificial Intelligence |
|  | 21 | AI |
|  | 22 | Big Data |
|  | 23 | Machine Learning |
|  | 24 | Augmented Reality |
|  | 25 | AR |
|  | 26 | Virtual Reality |
|  | 27 | VR |

**Filters and Limits:**

- English Language
- No date range was set, the last database search was conducted on August 16^th^, 2022

**Final Boolean Search Strategy:**
*(TI privacy OR "privacy paradox" OR "privacy concern" OR “privacy concerns”)****AND*** *("privacy concern" OR "privacy concerns" OR "privacy paradox" OR personalization paradox OR perceived vulnerability OR disclosure OR perceived control OR risk OR willingness to disclose OR consumer OR user OR adoption)****AND*** *(internet of things OR iot OR "smart device" OR "smart devices" OR "connected device" OR "connected devices" OR "artificial intelligence" OR ai OR big data OR machine learning OR augmented reality OR ar OR virtual reality OR vr)*

***AND*** *(LANGUAGE: English)*
